# Supplementary material for: Resolving the full spectrum of human genome variation using Linked-Reads
Source: Genome Res. 2019 Apr;29(4):635–45. doi: 10.1101/gr.234443.118 (PMC6442396; doi:10.1101/gr.234443.118)
Supplement: Supplemental Material [file supp_gr.234443.118_Supplemental_longranger-2.2.zip › longranger-2.2.tar.bz2!/lr2.2-public/lib/go/src/code.google.com/vitess/docs/resources/percona-2016.pdf]

Vitess

The Complete Story

Percona Live Data Performance Conference

April 20, 2016

Sugu Sougoumarane, Anthony Yeh

http://vitess.io

What is Vitess?

Flipkart

"Flipkart's developers might soon forget what MySQL sharding is, thanks to Vitess."

Jolt

"After researching many different sharding strategies and tools used by the biggest companies we could find, Vitess was the obvious choice."

PIXELFEDERATION

"With Vitess, it is possible to shard your application data out of the box, with not more than 10 changed lines of code!"

YouTube

"We had to come up with something that would leap ahead of the curve instead of just fighting fires. When we finally built the initial feature list, it was obvious that we were addressing problems that are common to all growing organizations."

Outline

- Vitess Overview
  - Cluster Architecture
  - Client Libraries
  - Sharding Demo
- V3 API Deep Dive
  - Evolution
  - Concepts
  - Example

Vitess Overview

Encapsulated MySQL Scalability

Built on proven technologies:

- MySQL Replication
- InnoDB

Using proven techniques:

- Shared-nothing shards
- Consistent hashing

And proven at scale:

- Continuously deployed at YouTube (1B+ users)
- Thousands of DB servers, one oncall

Encapsulation means:

- The app is hidden from Vitess
  - Nothing YouTube-specific
- Sharding is hidden from the app
  - Looks like one logical DB
- Complexity is hidden from the operator
  - Operator overhead is O(1) as number of servers grows
- Maintenance is hidden from end users
  - No user-visible downtime

The Secret Vitess Master Plan

✅ Phase 1

YouTube never has to worry about DB scalability again.

➡ Phase 2

Anyone can run MySQL at YouTube scale in the cloud.

Phase 3

More workloads move into the cloud. ($$$)

What's New

Client Libraries

- Java (JDBC)
- PHP (PDO)
- Python (PEP 0249)
- Go (database/sql)

Query Support

- Cross-shard auto-increment
- Cross-shard joins
- Automatic shard lookup tables (secondary vindexes)

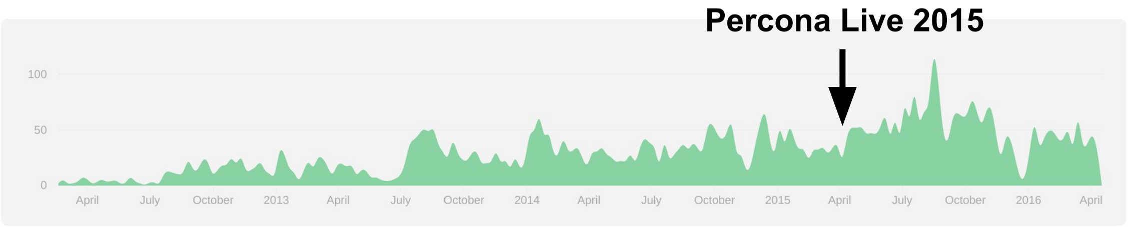

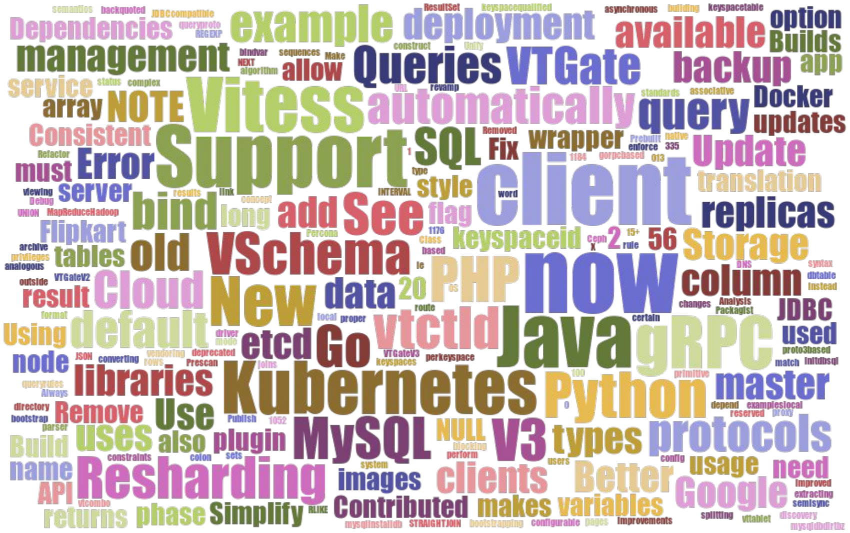

Scorecard: Last year's Roadmap slide

- MySQL 5.6 ✓
- Benchmarks (YCSB) ✓
- VTGate V3 ✓
  - Automatic lookup tables ✓
  - Cross-shard auto_increment ✓
  - MySQL binary protocol
- High-level automation
  - One-click resharding ✓
  - Rolling (OOB) schema changes
- More documentation
  - Architecture / design ✓
  - How-to guides ✓
- Kubernetes
  - API server proxy for debug access ✓
  - Replication controllers for tablets
  - Cloud plugins for backup/restore (Google Cloud Storage) ✓
- More client support
  - gRPC ✓
  - PHP ✓
- Long-term
  - Cross-shard transactions (2PC)
  - Cross-shard joins/aggregation

✓

Cluster Architecture

Components

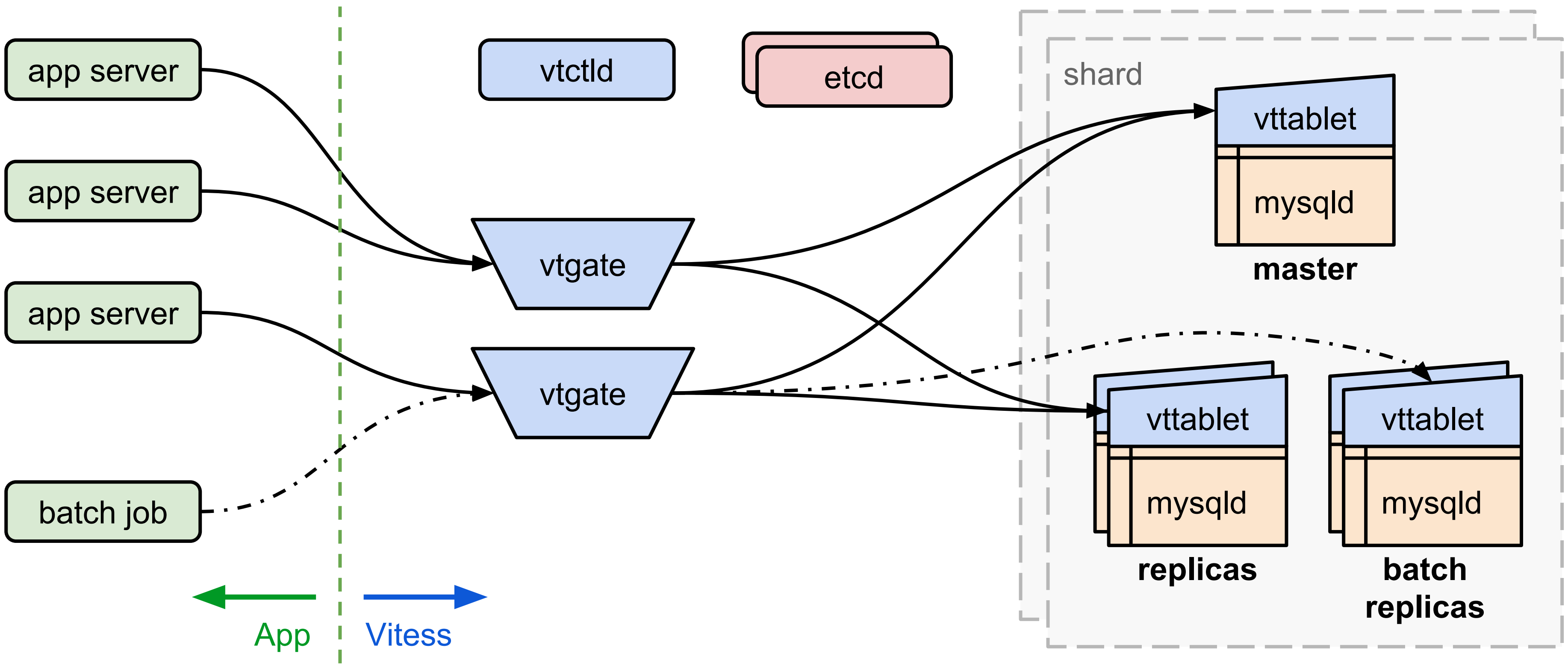

app server
app server
app server
batch job
vtctld
etcd
vtgate
vtgate
shard
vttablet
mysqld
master
vttablet
mysqld
replicas
vttablet
mysqld
batch
replicas
App
Vitess

Topology

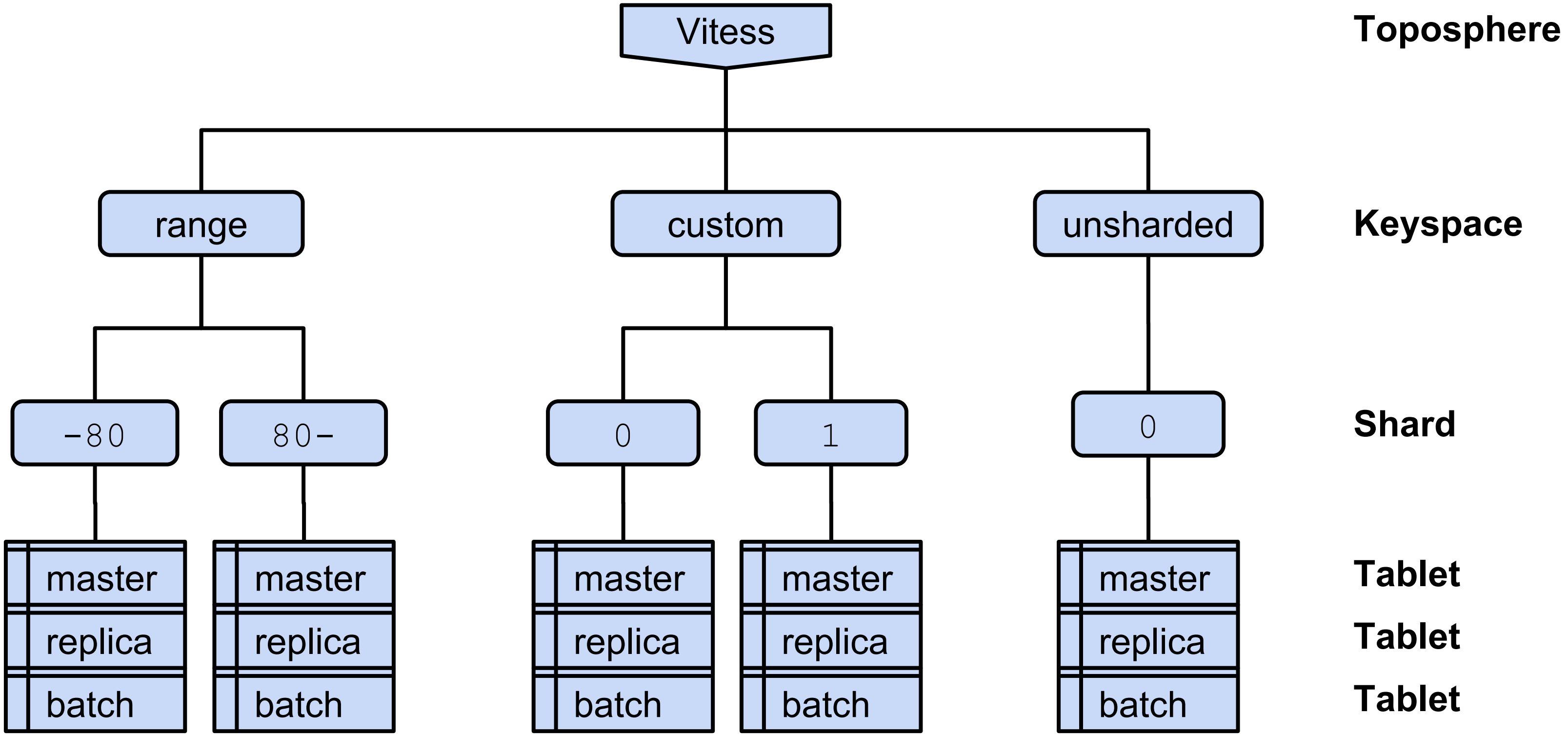

Vitess
range
custom
unsharded
-80
80-
0
1
0
master
replica
batch
master
replica
batch
master
replica
batch
master
replica
batch
master
replica
batch
Toposphere
Keyspace
Shard
Tablet
Tablet
Tablet

Consistent Hashing

- Keyspace ID: hash of sharding key (e.g. user ID)
- Shard Name: `[start]-[end]`
  - `keyspace_id, [start]` and `[end]` are all byte arrays
  - Compare byte arrays lexicographically (string compare):
    - **`[start] <= keyspace_id < [end]`**
  - Leave out `[start]` or `[end]` to make that side unbounded

Shard Name	Key Range (assuming 64-bit keyspace IDs)
`-80`	`00 00 00 00 00 00 00 00 - 7F FF FF FF FF FF FF FF`
`80-`	`80 00 00 00 00 00 00 00 - FF FF FF FF FF FF FF FF`

Bonus Question: What's the first shard name if you want 512 equal shards?

Hint: What's the first key range for 256 shards? What's the midpoint of that range?

Client Libraries

Java

- JDBC-compatible interface
  - Contributed by Flipkart
  - https://github.com/youtube/vitess

```
Connection conn =
    DriverManager.getConnection("jdbc:vitess://vtgate:15991/keyspace", null);
String sql = "select * from test_table where id = ?";
PreparedStatement preparedStatement = conn.prepareStatement(sql);
preparedStatement.setInt(1, 10);
rs = preparedStatement.executeQuery();
```

PHP

- PDO-compatible interface
  - Open-sourced by Pixel Federation
  - https://github.com/pixelfederation/vitess-php-pdo

```
$pdo = new PDO("vitess:dbname=keyspace;host=vtgate;port=15991");
$stmt = $pdo->prepare("SELECT * FROM user WHERE user_id IN (?, ?)");
$result = $stmt->execute([151, 152]);
```

Python

- PEP 249-compatible interface
  - https://github.com/youtube/vitess/blob/master/examples/kubernetes/guestbook

```
conn = vtgate_client.connect('grpc', 'vtgate:15991', timeout)
cursor = conn.cursor(tablet_type='replica', keyspace='keyspace')
cursor.execute('SELECT message, time_created_ns FROM messages WHERE page=:page
ORDER BY time_created_ns', {'page': page})
entries = [row[0] for row in cursor.fetchall()]
```

Go

- database/sql driver
  - https://godoc.org/github.com/youtube/vitess/go/vt/vitessdriver

```
db, err := vitessdriver.Open("vtgate:15991", "master", timeout)
tx, err := db.Begin()
_, err := tx.Exec("INSERT INTO test_table (msg) VALUES (?)", "V is for speed")
err := tx.Commit()
```

Sharding Demo

vitess.io/getting-started

vitess.io/user-guide/sharding-kubernetes.html

Guestbook

http://vitess.ddns.net

Guestbook Page 62

SUBMIT

http://vitess.ddns.net/page/62

/env

Kubernetes

kubernetes

← guestbook

PODS EVENTS

Replication controller

Name
guestbook

Namespace
default

Pods
10 running

Label selector
app: guestbook component: frontend

Labels
app: guestbook component: frontend

Images
vitess/guestbook

Services

Name
guestbook

Label selector
app: guestbook component: frontend

Internal endpoint
guestbook:80 TCP

External endpoint
104.197.29.52:80

↑ Pod	Status	Restarts	Age	CPU (cores)	Memory (B)	Cluster IP	Node	Logs
guestbook-001ny	Running	1	20 minutes	0.004	60.883 Mi	10.64.2.5	gke-example-default-pool-013ab373-f4it	Logs
guestbook-88bfy	Running	1	20 minutes	0.004	69.289 Mi	10.64.2.4	gke-example-default-pool-013ab373-f4it	Logs
guestbook-8hn3p	Running	1	20 minutes	0.005	79.031 Mi	10.64.4.8	gke-example-default-pool-013ab373-79tg	Logs
guestbook-kgeby	Running	8	3 days	0.005	85.828 Mi	10.64.3.3	gke-example-default-pool-013ab373-k3v6	Logs
guestbook-pw0mx	Running	1	20 minutes	0.004	58.531 Mi	10.64.0.8	gke-example-default-pool-013ab373-fpvz	Logs
guestbook-py4s8	Running	7	4 days	0.005	116.414 Mi	10.64.1.8	gke-example-default-pool-013ab373-h3k2	Logs
guestbook-sb8j3	Running	8	4 days	0.004	65.477 Mi	10.64.0.6	gke-example-default-pool-013ab373-fpvz	Logs
guestbook-sggnh	Running	1	20 minutes	0.005	68.773 Mi	10.64.3.7	gke-example-default-pool-013ab373-k3v6	Logs
guestbook-sw5z2	Running	1	20 minutes	0.004	81.398 Mi	10.64.2.6	gke-example-default-pool-013ab373-f4it	Logs
guestbook-zbno3	Running	1	20 minutes	0.004	66.656 Mi	10.64.2.7	gke-example-default-pool-013ab373-f4it	Logs

vtctld

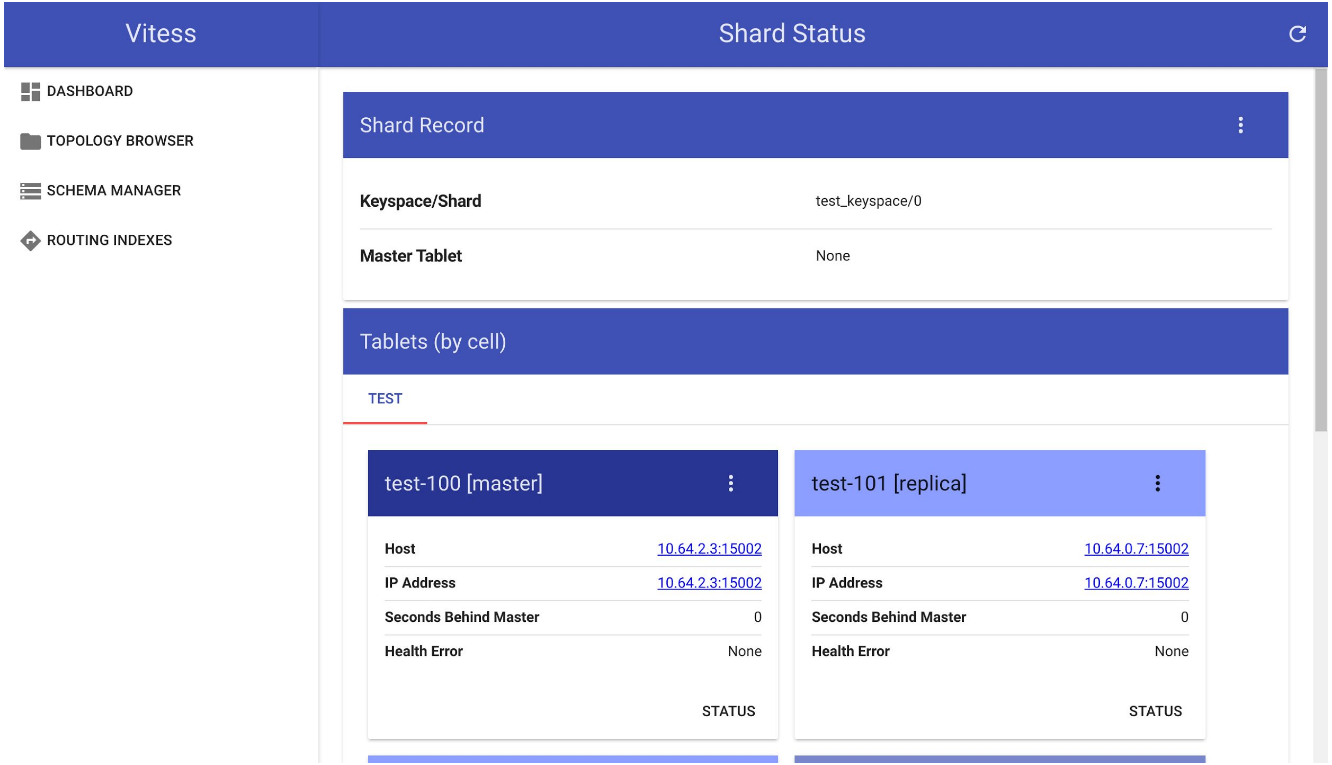
Vitess
Shard Status
DASHBOARD
TOPOLOGY BROWSER
SCHEMA MANAGER
ROUTING INDEXES
Shard Record
Keyspace/Shard
test_keyspace/0
Master Tablet
None
Tablets (by cell)
TEST
test-100 [master]
Host
10.64.2.3:15002
IP Address
10.64.2.3:15002
Seconds Behind Master
0
Health Error
None
STATUS
test-101 [replica]
Host
10.64.0.7:15002
IP Address
10.64.0.7:15002
Seconds Behind Master
0
Health Error
None
STATUS

vttablet

Status for vttablet

Started: Wed, 20 Apr 2016 06:20:09 UTC

Running on vttablet-100
View variables, debugging profiles,

Tablet

Alias: test-0000000100<br>Keyspace: test_keyspace Shard: 0<br>Serving graph: test_keyspace 0 master<br>Replication graph: test/test_keyspace/0	Schema<br>Schema Query Plans<br>Schema Query Stats<br>Schema Table Stats	Query Stats<br>Consolidations<br>Current Query Log<br>Current Transaction Log	Health Check<br>Query Service Health Check<br>Memcache<br>Current Stream Queries

Health

Current status: healthy

Polling health information from . (healthCheckInterval: 5s; degradedThreshold: 30s; unhealthyThreshold: 2h0m0s)

Health History

Time	Healthcheck Result
Apr 20, 2016 at 06:24:07 (UTC)	healthy
Apr 20, 2016 at 06:21:01 (UTC)	unhealthy: Unknown database 'vt_test_keyspace' (errno 1049)
Apr 20, 2016 at 06:20:16 (UTC)	unhealthy: Can't connect to local MySQL server through socket '/vt/vtdataroot/vt_0000000100/mysql.sock' (2) (errno 2002)

healthy
: serving traffic.

unhappy
: will serve traffic only if there are no fully healthy tablets.

unhealthy
: will not serve traffic.

Queryservice

Sharding Information

Schema

```
CREATE TABLE messages (
  page BIGINT(20) UNSIGNED,
  time_created_ns BIGINT(20) UNSIGNED,
  message VARCHAR(10000),
  PRIMARY KEY (page, time_created_ns)
) ENGINE=InnoDB
```

VSchema

```
{
  "Sharded": true,
  "Vindexes": { "hash": {"Type": "hash"} },
  "Tables": {
    "messages": {
      "ColVindexes": [
        {"Col": "page", "Name": "hash"}
      ]
    }
  }
}
```

Live Migration

Old and new shards overlap during migration.

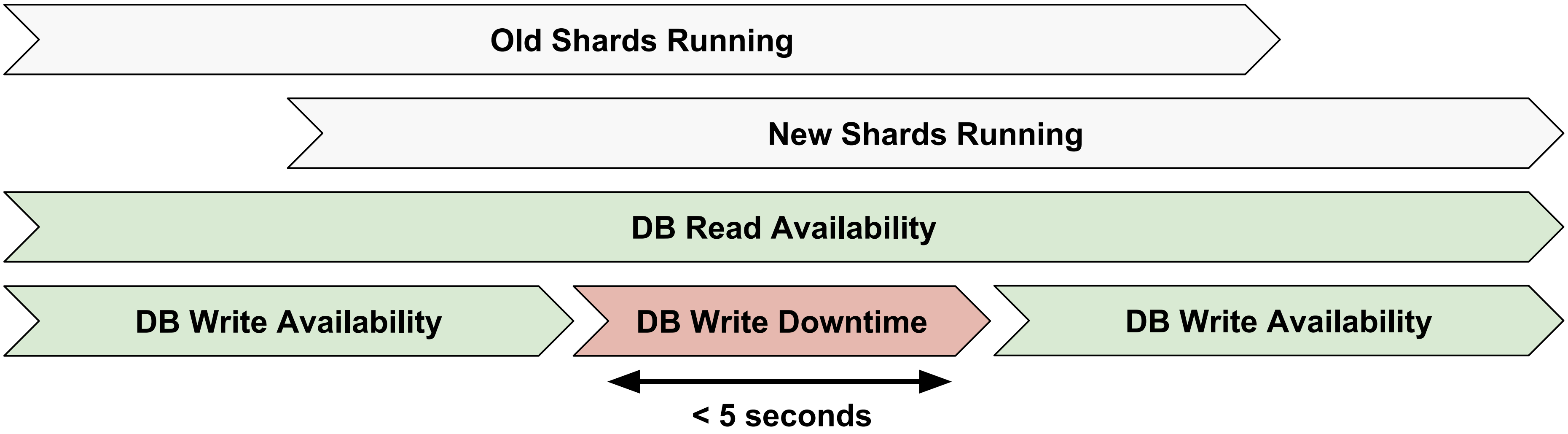

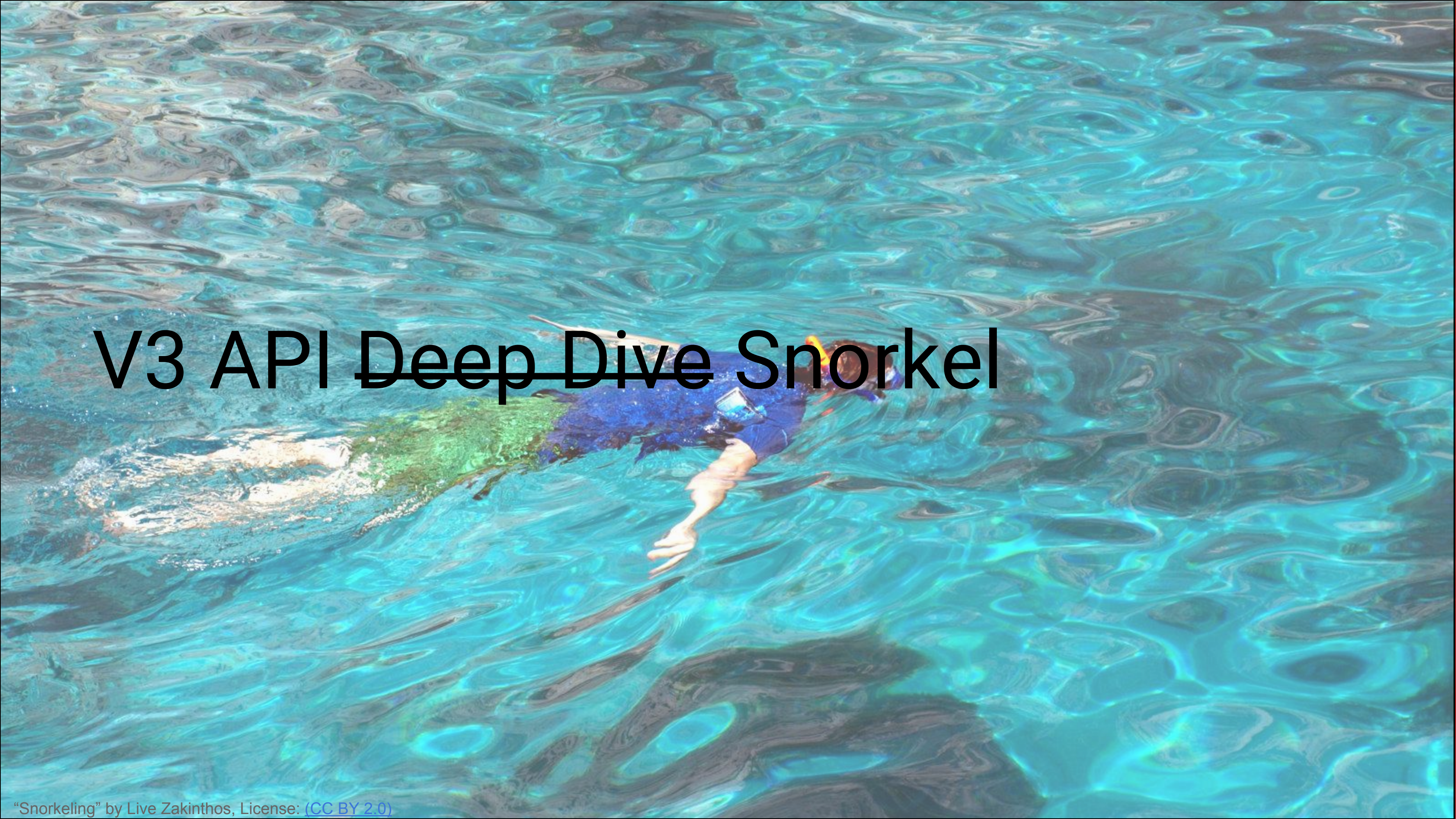

“Snorkeling” by Live Zakinthos, License: (CC BY 2.0)

Evolution

V0

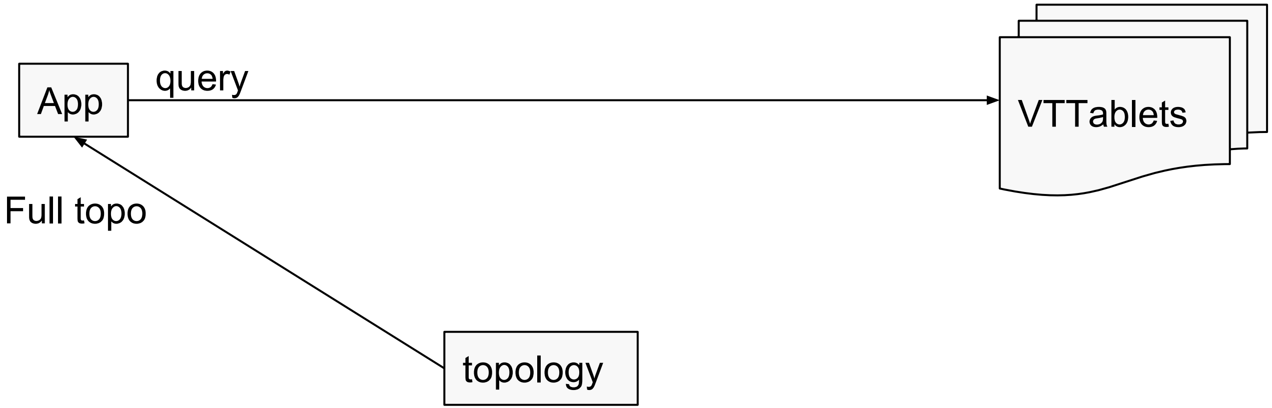

V1

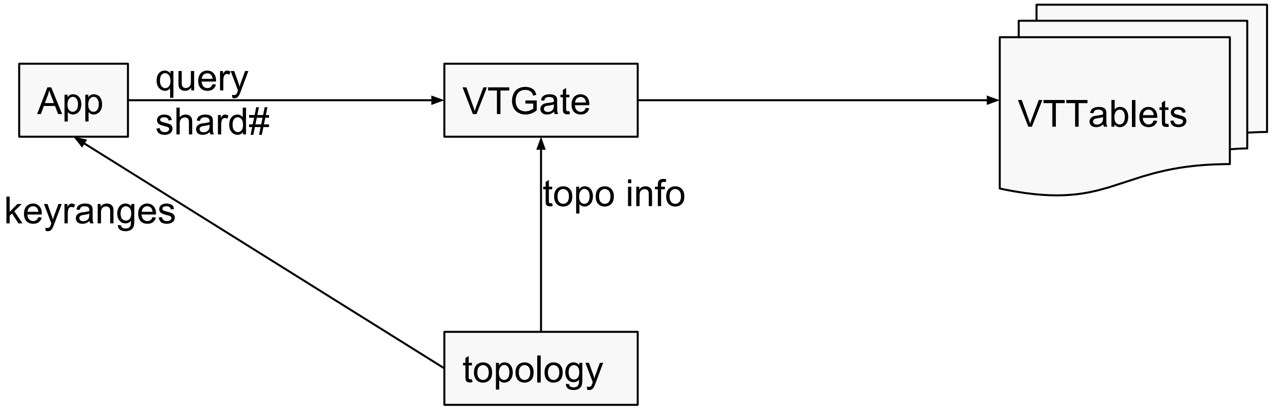
App
query
shard#
VTGate
VTTablets
keyranges
topo info
topology

V2

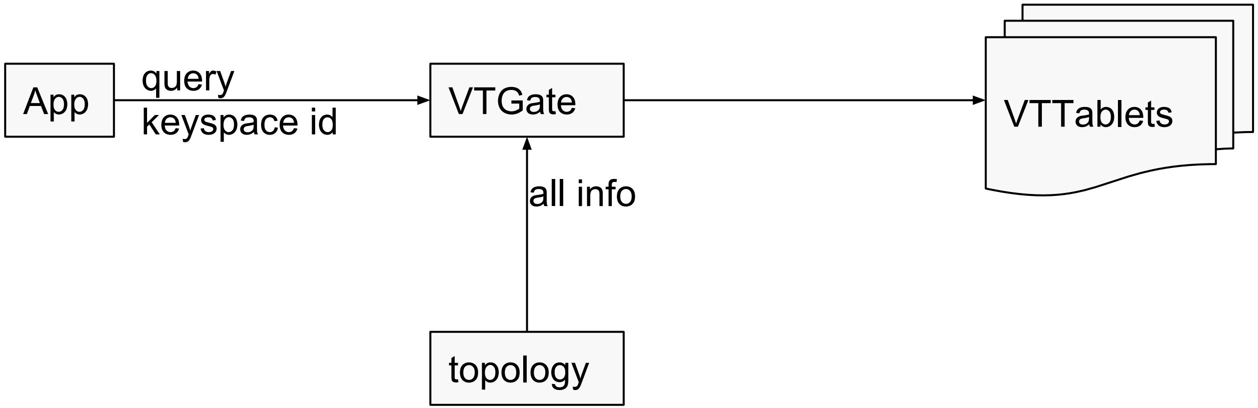

V3

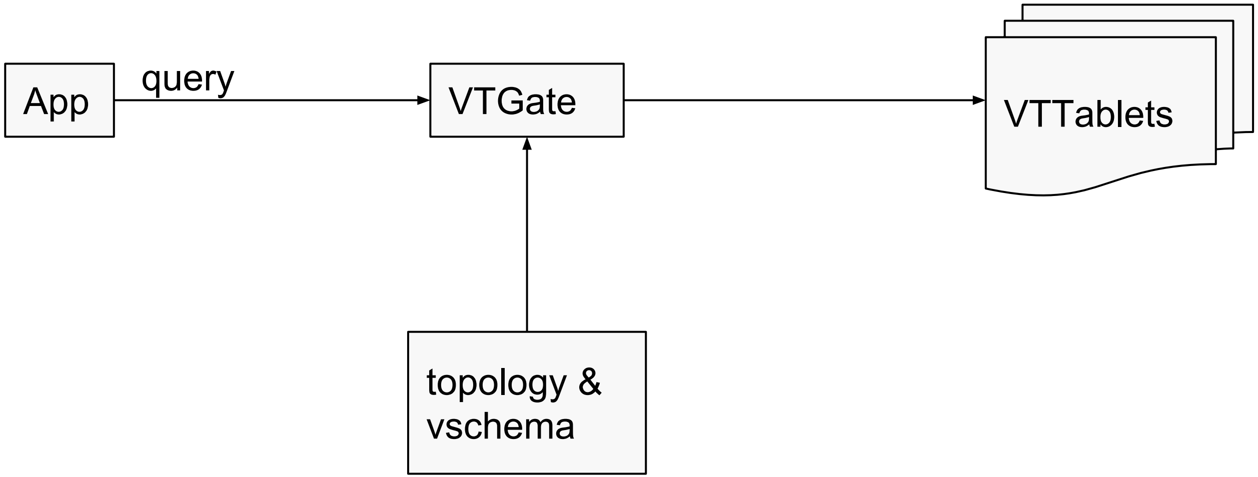

Game changer

- Drop-in replacement*
- Feel like a unified database
- DB-compliant APIs
  - Third party tools can integrate
- Sharding key can be an afterthought

* mostly

Concepts

What’s an index

- Enforces constraints
- Lets you scan a subset of the rows

- An index is a table
- A table can act as an index

Vindexes (Vitess Indexes)

- Analogous to SQL indexes
- Output of a Vindex is one or many keyspace ids
- May be Unique or NonUnique
- A Primary Vindex must be Unique
- A Vindex can be Functional or Lookup
- A Vindex can be user-defined
  - Pluggable sharding scheme

Index vs Vindex

**SQL**	**Vitess**
Row ID (not in MySQL)	Keyspace ID
Primary Key	Primary Vindex (usually the sharding key)
Secondary index	Lookup Table (cross-shard index)
Foreign Key	Shared Lookup Table
Auto-inc	Sequences
	Custom Index (pluggable sharding)

Vindex usage

- Routing: Where should a query be sent
- Sharding: Where should a row be sent
  - No explicit designation of sharding key
  - Works equally well with user-defined vindexes

What’s a table

- A SELECT can stand-in for a table
- A database can serve SELECTs
- A database can be treated like a table

Parallels

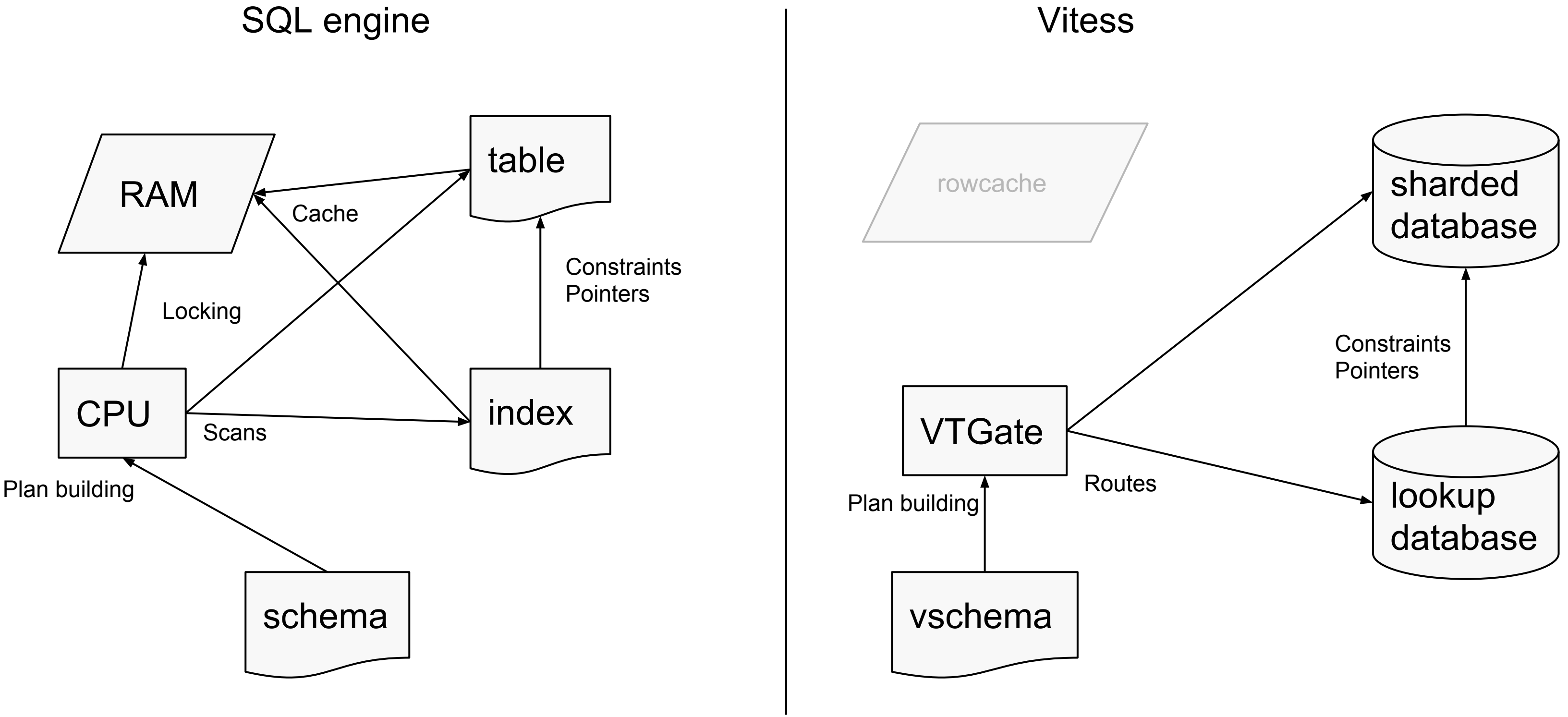
SQL engine
RAM
table
Cache
Constraints
Pointers
Locking
CPU
index
Scans
Plan building
schema
Vitess
rowcache
sharded
database
Constraints
Pointers
VTGate
Routes
Plan building
lookup
database
vschema

Some differences

- Network hops are much more expensive
- Each node is versatile

- Full databases underneath, instead of dumb tables
- Entire queries (or chunks) can be outsourced
- Results can still be combined like a traditional engine
- VTGate is both an engine and a router

Example

https://github.com/.../examples/demo

Schema

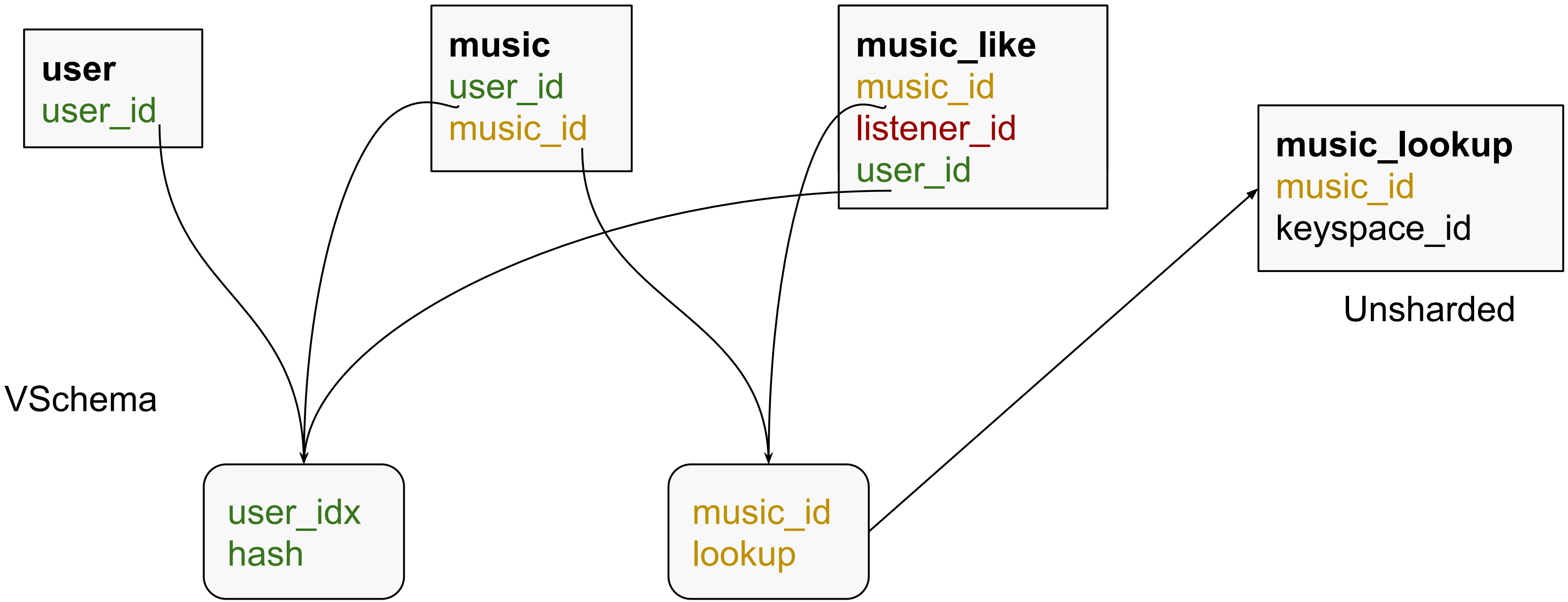
user
user_id
music
user_id
music_id
music_like
music_id
listener_id
user_id
music_lookup
music_id
keyspace_id
Unsharded
VSchema
user_idx
hash
music_id
lookup

Queries

- select * from user where user_id=5
- select * from user where user_id in (1, 5)
- select * from music where user_id=5
- select * from music where music_id=2
- select * from music_like where music_id=2
- select * from user

Joins and subqueries

- Treat databases as tables
- Outsource as much as possible to the databases
- Do only wire-up work in VTGate

Join queries

- select u.name, m.song from user u join music m on u.user_id=m.user_id where user_id=5
- select m.song, u.name from music m join music_like ml on m.music_id=ml.music_id join user u on ml.listener_id=u.user_id where m.music_id=2
  - select m.song, ml.listener from music m join music_like ml on m.music_id=ml.music_id where m.music_id=2
  - select u.name from user u where u.user_id=:ml_listener
- select u1.name, u2.name from user u1 join user u2 on u1.user_id = u2.fan_id

Current limitations

- Cross-shard post-processing
- Cross-shard subqueries

- Any work that cannot be outsourced (other than joins)

Distributed transactions

“If a group of transactional engines provide a certain durability guarantee, then it’s possible to extend the same guarantee to distributed transactions that span those engines”

Exclusions

- Commit should be non-failable
- No internal errors
- No isolation guarantees

The End...?

Automated Master Election

- Orchestrator
  - by Shlomi Noach (GitHub, formerly Booking.com)
  - github.com/outbrain/orchestrator

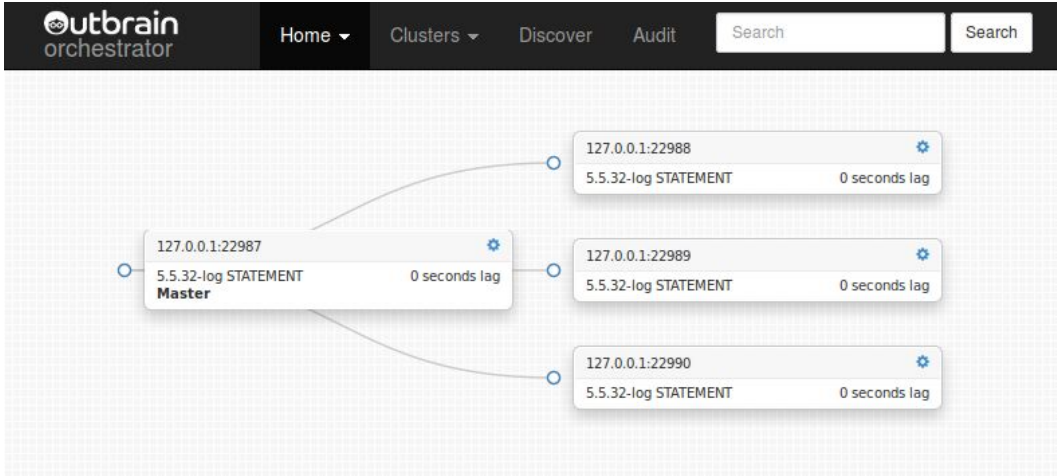

Automated Background Schema Rollout

- For schema changes that are too slow to replicate
- Options
  - pt-online-schema-change
  - https://github.com/square/shift
  - Google MySQL "Pivot"

Resources

Try Vitess

vitess.io/getting-started

Contribute

github.com/youtube/vitess

Contact Us

vitess@googlegroups.com

Get Updates

groups.google.com/d/forum/vitess-announce

blog.vitess.io

Cloud Native Computing Foundation

cncf.io

Kubernetes

kubernetes.io

Extra Slides

Join example

select a.id, a.a1, b.b1 from a join b on a.n = b.n where a.id = 5

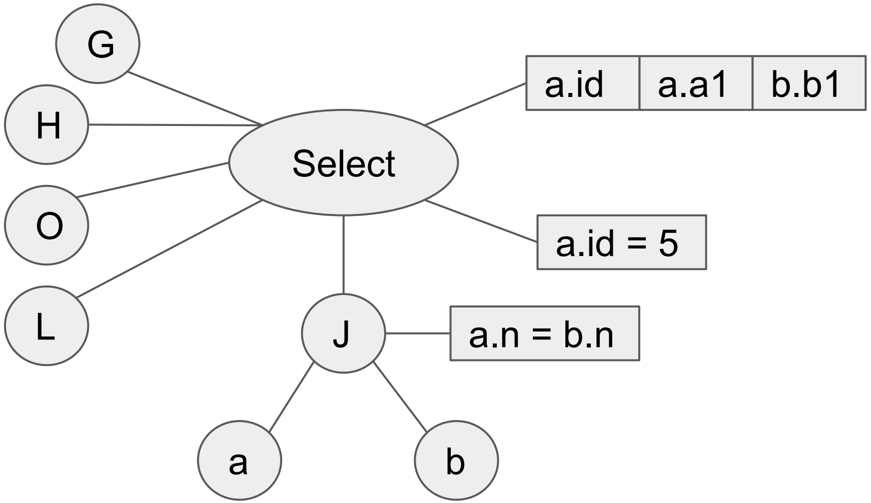

Build plan

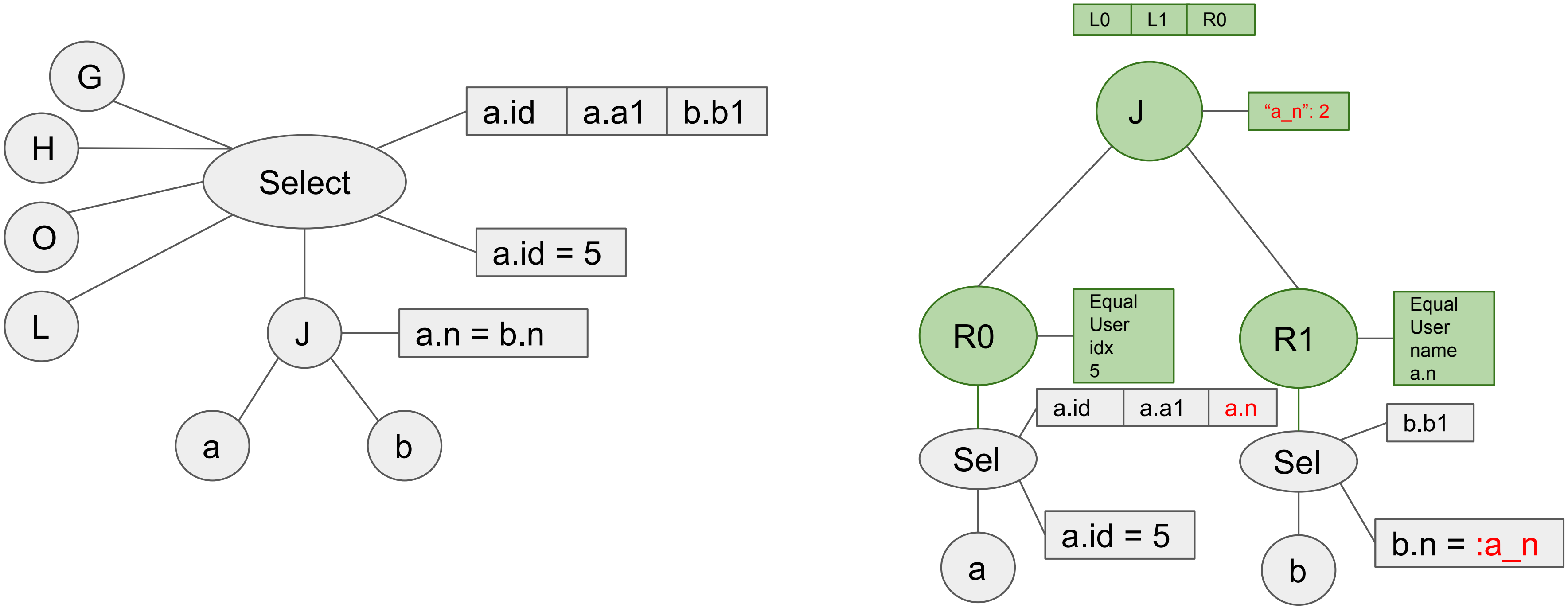

G
H
O
L
Select
a.id
a.a1
b.b1
a.id = 5
J
a.n = b.n
a
b
L0
L1
R0
J
“a_n”: 2
R0
Equal
User
idx
5
R1
Equal
User
name
a.n
a.id
a.a1
a.n
b.b1
Sel
Sel
a.id = 5
b.n = :a_n
a
b

Finalize plan

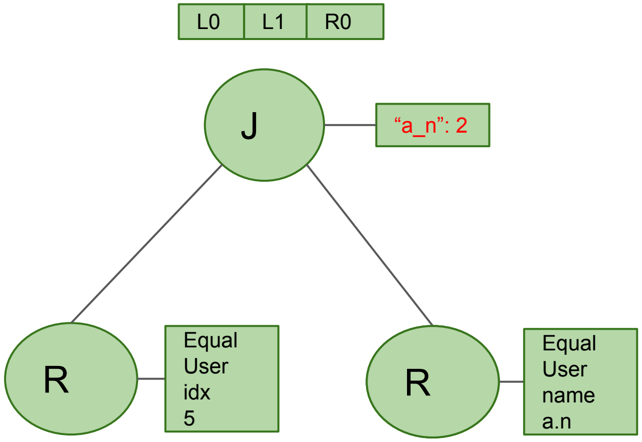

select a.id, a.a1, a.n
from a
where a.id = 5

select b.b1
from b
where b.n = :a_n
